# Cardiac Restoration Stemming From the Placenta Tree: Insights From Fetal and Perinatal Cell Biology

**DOI:** 10.3389/fphys.2018.00385

**Published:** 2018-04-11

**Authors:** Sveva Bollini, Antonietta R. Silini, Asmita Banerjee, Susanne Wolbank, Carolina Balbi, Ornella Parolini

**Affiliations:** ^1^Regenerative Medicine Laboratory, Department of Experimental Medicine, University of Genova, Genova, Italy; ^2^Centro di Ricerca E. Menni, Fondazione Poliambulanza - Istituto Ospedaliero, Brescia, Italy; ^3^Ludwig Boltzmann Institute for Experimental and Clinical Traumatology, AUVA Research Center Austrian Cluster for Tissue Regeneration, Vienna, Austria; ^4^Institute of Human Anatomy and Cell Biology, “A. Gemelli” Faculty of Medicine and Surgery, Catholic University of the Sacred Heart, Rome, Italy

**Keywords:** amniotic fluid, placenta, umbilical cord, cardiac repair, cardiomyocyte, immunomodulation, cardioprotection, paracrine effect

## Abstract

Efficient cardiac repair and ultimate regeneration still represents one of the main challenges of modern medicine. Indeed, cardiovascular disease can derive from independent conditions upsetting heart structure and performance: myocardial ischemia and infarction (MI), pharmacological cardiotoxicity, and congenital heart defects, just to name a few. All these disorders have profound consequences on cardiac tissue, inducing the onset of heart failure over time. Since the cure is currently represented by heart transplantation, which is extremely difficult due to the shortage of donors, much effort is being dedicated to developing innovative therapeutic strategies based on stem cell exploitation. Among the broad scenario of stem/progenitor cell subpopulations, fetal and perinatal sources, namely amniotic fluid and term placenta, have gained interest due to their peculiar regenerative capacity, high self-renewal capability, and ease of collection from clinical waste material. In this review, we will provide the state-of-the-art on fetal perinatal stem cells for cardiac repair and regeneration. We will discuss different pathological conditions and the main therapeutic strategies proposed, including cell transplantation, putative paracrine therapy, reprogramming, and tissue engineering approaches.

## Healing a broken heart: a main challenge in regenerative medicine

Cardiovascular disease and heart failure are the main killers in the Western countries representing a significant economic burden for the national health systems, as more than 1 million hospitalizations are annually reported in the EU alone. Indeed, for the majority of people suffering from heart failure, the mortality rates remains high, with 1 out of 4 patients dying within 1 year from diagnosis (Jameel and Zhang, 2009). Despite significant efforts, current therapies for cardiovascular disease have not yet fulfilled all expectations, being intrinsically non-curative and with limited improvements in reducing mortality or delaying the onset of heart failure. Currently, the ultimate therapy is still represented by organ transplantation, mainly confounded by limited supply of donor organs (Levy et al., 2002). Several different conditions can affect the cardiac tissue, from ischemic disease to drug-derived cardiotoxicity up to congenital heart defects, overall resulting in pathological disruption of heart function with the main challenge to overcome mostly represented by primary loss of cardiomyocytes.

Nonetheless, a major breakthrough for cardiac regenerative medicine has been provided by mounting evidence suggesting that the heart retains an endogenous regeneration programme, although very limited, based on cardiac progenitor cell (CPC) activation and cardiomyocyte proliferation. Unfortunately, while broadly active in the neonate, these mechanisms are quiescent and malfunctioning in the adult heart (Porrello et al., 2011; Jesty et al., 2012). Therefore, a working scheme to fully unlock and rejuvenate this potential will open new frontiers in cardiac medicine. Likewise, cardiomyocyte replacement and new vasculature formation by pluripotent-derived cell therapy, tissues engineering, and local cell reprogramming have also been widely scrutinized to identify an innovative therapeutic approach.

In particular, the optimal regenerative strategy should consider the specific patient clinical need and the pathological setting to operate within, in order to induce efficient tissue regeneration.

Myocardial infarction (MI) is the most common cause of cardiac injury in Western countries. Modern clinical interventions have substantially decreased acute mortality of MI, but have only partially diminished the number of patients who subsequently suffer from impairment of cardiac function, up to overt heart failure. The milieu of the injured heart is a battlefield for regeneration. In particular, after ischemic injury vascular supply is limited. Matrix stiffening that occurs after injury can further suppress the already limited potential for cardiomyocyte proliferation (Tzahor and Poss, 2017). Hence, prompt intervention is required to: (i) provide cardioprotection, modulate the inflammatory response, and improve wound healing, while (ii) activating resident CPC to differentiate and/or release trophic factors to support the microenvironment, and (iii) sustain proliferation of surviving cardiomyocytes, overall improving cardiac function. In such scenario, timely intervention is required and enormous interest has recently been driven toward stem/stromal cell biology for the regeneration of damaged tissues. The current endeavor of cell-based therapy in cardiac regenerative medicine is to stimulate endogenous restoration mechanisms; hence, growing interest has been directed toward speculative paracrine therapy based on exploiting the stem cell *secretome*, i.e., the totality of soluble paracrine factors and microRNA (miRNA)-enriched extracellular vesicles secreted by stem cells with beneficial effects on the injured myocardial tissue (Rani et al., 2015). Indeed, the MI patient presented at the hospital emergency room could be treated with the optimal drug formulation of the ideal pro-regenerative stem cell secretome to be delivered by intracoronary infusion during standard angioplasty procedure.

Cardioprotection from chemotherapy-derived cardiotoxicity represents another relevant clinical need that may be addressed by exploiting stem cell modulatory paracrine potential. Cardiotoxicity-induced cardiomyopathy leading to subclinical ventricular dysfunction or symptomatic heart failure can represent a critical complication as a side effect of oncological therapy. Notably, oncological treatments can affect both cardiomyocytes and endogenous CPC, leading to their senescence and/or apoptosis (Huang et al., 2010; Su et al., 2015). In this scenario, prompt modulation of the cardiac microenvironment could represent a crucial step to counteract chemotherapy-derived unspecific and noxious side effects and avoid long-term cardiovascular complications in cancer survivors (Hahn et al., 2014; Giza et al., 2017; Naaktgeboren et al., 2017). Indeed, a suitable preventive and complementary treatment during oncological therapy could be represented by the administration of stem cell-based secretome drug formulation via catheter-guided coronary infusion prior to chemotherapy, in order to ensure cardiac-specific delivery without jeopardizing oncological therapy.

Last, but not least, myocardial renewal for congenital heart defects is another major challenge in cardiac regenerative medicine. Congenital heart defects are common birth deficiencies often resulting in pediatric heart failure. They range from life-threatening conditions (i.e., hypo-plastic left heart syndrome) to benign defects (i.e., septal defects). CHD represent a complicated scenario with the explicit demand of reconstituting tissue that might have gone missing during development or have not properly formed (Woodward, 2011; Sun et al., 2015). Standard therapy is elective surgery within the first weeks of life to provide structural reconstruction using prosthetic implants (Ohye et al., 2016) that do not grow with the patient, thus requiring additional surgeries during life. Therefore, a suitable therapeutic approach should provide new functional cardiovascular and cardiac cells via stem cell-based therapy combined with tissue engineering strategies. Indeed, cardiovascular cells obtained by pluripotent stem cells could be loaded on biocompatible prosthetic material implanted during surgical interventions and sustain post-natal cardiac development.

In this review we will discuss the promising therapeutic role of non-embryonic fetal stem cells, from the amniotic fluid and placenta-derived progenitors in addressing relevant critical clinical needs for future cardiac regenerative medicine.

## Fetal progenitors: amniotic fluid stem cells for cardiac repair and regeneration

The human amniotic fluid contains different cell subpopulations with heterogenous phenotypes (epithelioid, “amniotic” and fibroblastic type) deriving from the developing fetus, including mesenchymal broadly multipotent progenitors endowed with high self-renewal and clonogenic potential, as illustrated by several studies (In ‘t Anker et al., 2003; Prusa et al., 2003; De Coppi et al., 2007; Pozzobon et al., 2013). Immature amniotic fluid-derived stem cells can be easily isolated from leftover back-up sample for prenatal screening via amniocentesis or amnio-reduction or at term, from discarded amniotic fluid obtained as clinical waste during scheduled cesarean delivery (Schiavo et al., 2015; Loukogeorgakis and De Coppi, 2017). Human amniotic fluid stem cells can be mainly divided into two categories: mesenchymal progenitors isolated by their intrinsic capacity to adhere to the culture dish, namely amniotic fluid-derived mesenchymal stem cells (AF-MSC) and a further subpopulation which can be specifically identified by the expression of the stem marker c-KIT via FACS- or immuno-magnetic sorting (hAFSC, Ditadi et al., 2009; Pozzobon et al., 2013); moreover, amniotic fluid-derived stem cells have shown to possess osteogenic, myogenic, and adipogenic potentials similar to mesenchymal stem cells (De Coppi et al., 2007).

In recent years several independent studies have scrutinized the cardiovascular and cardiomyogenic potential of stem cells from amniotic fluid as an appealing tool for therapeutic cellular cardiomyoplasty for heart regeneration following ischemic injury (Chiavegato et al., 2007; Iop et al., 2008; Walther et al., 2009; Yeh et al., 2010; Bollini et al., 2011a). Being immature fetal progenitors, AF-MSC and hAFSC were initially considered more prone to commit to specific cardiac and cardiovascular lineages, upon suitable stimulation. Nevertheless, while amniotic fluid stem cells can easily adopt a smooth muscle and/or endothelial fate both *in vitro* and *in vivo* (Sartore et al., 2005; Iop et al., 2008; Bollini et al., 2011a; Ghionzoli et al., 2013; Schiavo et al., 2015; Tancharoen et al., 2017), a general consensus on their cardiomyogenic potential has not been reached yet. AF-MSC and hAFSC have shown to acquire cardiomyocyte-like phenotype following specific *in vitro* treatment (i.e., via direct co-culture with rodent neonatal cardiomyocytes or chemical induction by 5-aza-2′-deoxycytidine, with or without the addition of transforming growth factor beta-1, or by a mixture of hyaluronic, butyric and retinoic acids, up to modulation of Wnt signaling by small molecules), with evidence including immature expression of sarcomeric proteins, like cardiac troponins, along with up-regulation of early cardiac transcription factors, such as Nkx-2.5, Islet-1 and Gata-4 (Chiavegato et al., 2007; Bollini et al., 2011a; Guan et al., 2011; Maioli et al., 2013; Gao et al., 2014; Connell et al., 2015; Jiang and Zhang, 2017). However, in most cases, no organized sarcomeres were detected in the differentiated cells (Connell et al., 2015), with limited spontaneous contraction or functional maturation of their phenotype (Bollini et al., 2011a). Likewise, when transplanted into preclinical pig and rodent models of myocardial infarction, AF-MSC and AFSC maintained their disposition toward the vascular lineages via angiogenic differentiation, but almost completely failed to trans-differentiate into functionally mature cardiomyocytes, providing questionable results (Sartore et al., 2005; Chiavegato et al., 2007; Bollini et al., 2011a; Lee et al., 2011). Therefore, despite the initial enthusiasm and great expectations, it is now quite clear that amniotic fluid stem cells may require extensive *ex-vivo* reprogramming to be suitable for therapeutic cardiomyoplasty.

Yet, despite the low grade of *in vivo* engraftment and differentiation of amniotic fluid stem cells transplanted into preclinical animal models of myocardial infarction, different studies reported improvement of cardiac function with higher vascular density, increased cardiomyocyte survival and attenuation of ventricular remodeling (Bollini et al., 2011b; Lee et al., 2011). These data clearly suggest that stem cell-secreted molecules can influence *in situ* cell-cell interactions, establishing a regenerative milieu in the injured microenvironment by paracrine effects. Indeed, there is strong evidence that crucial cellular functions such as survival, proliferation, differentiation, communication, and migration can be specifically orchestrated by the secretome of stem cells injected into the injured cardiac tissue (Gnecchi et al., 2006, 2008; Mirotsou et al., 2011). First evidence of hAFSC paracrine cardio-protective potential came from a study in 2011 from Bollini et al. (2011b), showing that systemic injection of cells vs. their conditioned medium (hAFSC-CM) into an acute rat model of myocardial ischemia/reperfusion injury equally improved cell survival and significantly decreased infarct size by about 14% in 2 h (Bollini et al., 2011b). As well, independent studies confirmed that hAFSC can evoke a strong angiogenic response in murine recipients and promote neo-arteriogenesis in preclinical rodent models of hind-limb ischemia and ischemic fascio-cutaneous flap, due to the remarkable paracrine potential of their secretome supplemented by MCP-1, IL-8, SDF-1, and VEGF (Mirabella et al., 2011, 2012).

More recently, a preconditioning cell culture protocol has been optimized based on a short burst of hypoxia under serum-free condition to enrich the hAFSC secretome with cardio-active soluble factors. The paracrine cardio-protective potential of the hypoxic hAFSC-CM has been tested *in vitro* in a doxorubicin-derived cardiotoxicity model, showing to effectively antagonize premature senescence and apoptosis of murine neonatal cardiomyocytes and human cardiac progenitor cells. Such paracrine modulation was demonstrated to act on responder cells via prompt activation of the PI3K/Akt signaling cascade, resulting in decreased DNA damage, nuclear translocation of NF-kB, and upregulation of the NF-kB controlled genes, Il6 and Cxcl1, which support cardiomyocyte survival. The hypoxic hAFSC-CM also showed to instruct cardiomyocytes to up-regulate the efflux transporter, Abcb1b, thus triggering active extrusion of the drug from cardiac cells (Lazzarini et al., 2016).

The first characterization of extracellular vesicles (EV) released by hAFSC, namely hAFSC-EV, has also been recently reported (Balbi et al., 2017; Mellows et al., 2017). EV, including microvesicles and exosomes, are membrane-enclosed micro- and nanovesicles constitutively shed by every cell; in particular stem cell-derived EV have been proposed to act as biological carrier of paracrine regenerative soluble factors, including microRNA and mRNA, into target cells (Kishore and Khan, 2016; Marote et al., 2016; Shafei et al., 2017). Thus, the stem cell-derived EV being safer, immunologically inert and easier to manipulate than cell-based products (O'Loughlin et al., 2012; Lai et al., 2013), they are currently under detailed investigation as innovative promising approach for future regenerative cell-free therapy. Notably, hAFSC-EV demonstrated to be key mediators of regenerative paracrine effects, including stimulation of cell proliferation and survival, with remarkable modulatory potential in decreasing skeletal muscle inflammation *in vivo*. In particular, hypoxic hAFSC-EV mediated significant regenerative effects on responding cells by targeting post-transcriptional regulating mechanism by horizontal reprogramming via direct miRNA transfer, including miR-210 and miR-199a-3p, which have been previously reported to provide significant cardioprotection (Barile et al., 2014), sustain angiogenesis (Alaiti et al., 2012; Zeng et al., 2014) and the reactivation of cardiomyocyte proliferation (Eulalio et al., 2012). Therefore, human amniotic fluid stem cells represent a valuable candidate for future advanced medicinal/pharmacological product to treat both chemotherapy-related cardiotoxicity and myocardial infarction, due to their powerful paracrine potential. Yet, since optimal cardiac regeneration should be achieved by obtaining myocardial reconstitution on top of enhancing cardiac repair mechanisms, further investigation is required to assess whether the hAFSC secretome can also boost endogenous CPC reactivation and trigger resident cardiomyocyte proliferation following injury.

Another significantly relevant scenario is also represented by hAFSC as a potential therapeutic component in cardiac tissue engineering applications for congenital defect repairs. With most congenital heart defects being diagnosed by prenatal screening in the second trimester (Yagel et al., 1997), amniotic fluid may represent a convenient, exploitable, and autologous source easily available for collection during pregnancy. Previous studies from Hoerstrup and co-authors have suggested employing either freshly isolated or cryopreserved human amniotic fluid stem cells selected for CD133 expression to engineer heart valve leaflet scaffolds based on biodegradable polymers, with encouraging results in terms of endothelial tissue formation and functional behavior (Schmidt et al., 2007, 2008); likewise, ovine amniotic fluid stem cells have been used for *in vitro* fabrication of tri-leaflet heart valves to be implanted prenatally and orthotopically sheep fetuses, showing valvular integrity and absence of thrombus formation a week after transplantation (Weber et al., 2012). More recently, tissue engineered vascular grafts have been obtained from tubular vessel-like shaped biocompatible scaffolds seeded with ovine amniotic fluid progenitors under dynamic conditions in a flow bioreactor system, demonstrating the technical feasibility of such approach (Weber et al., 2016).

Since the major scientific breakthrough of induced pluripotent stem cells (iPS) technology in 2006 (Takahashi and Yamanaka, 2006), growing interest has been dedicated toward exploiting direct reprogramming of somatic cells into pluripotent progenitors as functional source to derive contractile autologous cardiomyocytes (Lian et al., 2012). This has been recently endorsed as a working strategy to overcome the incomplete and poor yield of somatic stem cell cardiomyogenic trans-differentiation, which may jeopardize myocardial renewal and reconstitution. Hence, the quest is now on defining the most suitable cell to efficiently induce iPS from and since amniotic fluid stem cells are immature fetal progenitors endowed with some degree of instrinsic pluripotency and active expression of embryonic genes including OCT4, NANOG, and SOX2 (Loukogeorgakis and De Coppi, 2017), they represent an ideal candidate. Recent studies have reported that murine and human AFSC can be reprogrammed into iPS more easily than adult stem cells by applying either transgene-free approaches, like chemical defined conditions via stimulation with the histone deacetylase inhibitor valproic acid (Moschidou et al., 1953), as well as non-integrating methods by episomal plasmid, transposon system, sendai virus or mRNA delivery by lipofection (Jiang et al., 2016; Slamecka et al., 2016; Bertin et al., 2017; Velasquez-Mao et al., 2017); notably the obtained AFSC-iPS have been proven capable of functional cardiac differentiation, thus providing important impact for future cardiac regenerative therapy and specific relevance for the treatment of neonatal cardiac congenital disease (Jiang et al., 2016; Velasquez-Mao et al., 2017). Indeed, hAFSC can be effortlessly harvested during prenatal diagnosis, treated by gene therapy and iPS technology to derived healthy myocardial and cardiovascular cells to be then processed by tissue engineering approaches so to provide cardiac grafts developed in parallel with gestation and promptly implanted *in utero* or at birth.

Notably, while II trimester fetal hAFSC have been widely investigated, little is known about the regenerative capacity of III trimester perinatal hAFSC obtained at term from discarded amniotic fluid samples from eligible cesarean delivery. Indeed, III trimester amniotic fluid might represent a more abundant source of hAFSC. While recent work has showed that III trimester hAFSC are endowed with significant pro-angiogeneic action (Schiavo et al., 2015), a detailed, comprehensive differential characterization of their cardiac and cardiovascular potential has not yet been provided.

## Perinatal progenitors: placenta-derived stem cells for cardiac repair

### The human placenta

Upon attachment and invasion into the uterine wall, the embryonic trophoblast concomitant with the embryoblast start differentiating, and together with maternal endometrial transition forming the placental tissues. These tissues establish implantation, support the fetus and maintain pregnancy by orchestrating the maternal adaption. At full term, the placenta is a large discoid organ, 15–20 cm in diameter and 2–3 cm in thickness, weighing approximately 500–600 g. At this time, the placenta comprises tissues of maternal and fetal origin (Malina, 1993). At the maternal side, the basal plate, a thin layer of maternal decidua basalis is covering the cotyledons that are interspaced by grooves forming the decidua septa. The maternal plate is closely intertwined with the fetal, the chorionic plate that also comprises the vascular system of arteries and veins that feed the umbilical cord (Sadler et al., 2012). The innermost of these fetal membranes is the amnion, enclosing the amniotic fluid. Main functions of the placenta are the selective exchange of metabolic and gaseous products between maternal and fetal bloodstreams and its endocrine activity secreting more than 100 peptides and steroid hormones (Burton and Fowden, 2015). The placenta is further unique in its immuno-regulatory functions allowing maternal tolerance and support of the growing embryo/fetus throughout pregnancy (Mori et al., 2016; Vinketova et al., 2016).

This highly active organ has also been recognized as rich source of human progenitor cells, extracellular matrix and bioactive compounds. Human placenta is usually discarded after birth, and hence ethically uncontroversial, it is large in size yielding high amounts of easily accessible human tissue compounds. With the beginning of the twenty first century, researchers started to realize that cells of placental tissues show distinct stem cell qualities, such as expression of markers of pluripotency (Miki et al., 2007) and the potential to differentiate into lineages of all three germ layers (Kakishita et al., 2003; Takashima et al., 2004; Miki et al., 2005; Portmann-Lanz et al., 2006), as well as paracrine properties such as anti-inflammatory, antibacterial and anti-fibrotic activities, which recommend these cells for regenerative medicine and wound healing applications (Cargnoni et al., 2009, 2014; Lopez-Espinosa et al., 2009; Hong et al., 2010; De et al., 2011; Choi et al., 2013; Ricci et al., 2013; SantAnna et al., 2016).

### Cells isolated from different placental tissues

Derivation and cultivation of several cell types with reported stem/progenitor properties from human term placenta has been described (Bailo et al., 2004; Miki et al., 2005; Soncini et al., 2007). Cell populations can be derived from specific placental tissues and the nomenclature used is based on the consensus from the First *International Workshop on Placenta-Derived Stem Cells* (Parolini et al., 2008): amnion to obtain human amniotic epithelial cells (hAEC) and amniotic mesenchymal stromal cells (hAMSC), and chorionic mesoderm to obtain chorionic mesenchymal stromal cells (hCMSC), chorionic villi to obtain MSC and endothelial progenitor cells (Zhang et al., 2006; Rapp et al., 2012) or placental cotyledons (Sölder et al., 2012), but also maternal cells from the decidua, to obtain decidual stromal cells (Huang et al., 2009). In addition, MSC can be obtained from umbilical cord (hUCMSC, including Wharton's Jelly) (Troyer and Weiss, 2008; La Rocca et al., 2009), from the chorionic plate/trophoblast (hCpMSC), and from placenta tissue in toto (i.e., no specific compartment selected; PDMSC). Herein other placental MSC will be discussed as well, such Placenta-derived Adherent cells (PDA-001, Celgene Therapeutics), and PLacental eXpanded (PLX) mesenchymal-like adherent stromal cells (PLX-PAD, Pluristem Therapeutics Inc.). Noteworthy, in a substantial number of studies the purity or even identity of fetal or maternal origin of cells isolated from the placenta chorionic plate and decidua has not been investigated (Heazlewood et al., 2014).

### General features of placental cells

#### Phenotype

Several studies demonstrate that the adherent cellular fraction derived from placenta express cell surface markers similar to bone marrow-derived mesenchymal stem/stromal cells such CD13, CD29, CD44, CD73, CD90, CD105, CD166, and MHC I, and are mostly negative for CD14, CD34, CD45, and MHC II at term (In ‘t Anker et al., 2003; Portmann-Lanz et al., 2006; Parolini et al., 2008; Magatti et al., 2015). A sub-fraction of these cells have further been shown to express heterogeneous degrees of markers previously identified in pluripotent stem cells including SSEA-4, Tra-160, Tra-181, octamer-binding protein 4 (Oct-4), Nanog or Sox-2 (Miki and Strom, 2006; Portmann-Lanz et al., 2006; Alviano et al., 2007; Kim et al., 2007; Miki et al., 2007), the level of which however depends on factors such as the gestational age of the placenta (Izumi et al., 2009; Barboni et al., 2014).

#### Differentiation potential

It has been suggested that placenta-derived cells can be classified as an intermediate state between pluripotent stem cells and multipotent adult stem cells (reviewed by Kang et al., 2012). However, in contrast to embryonic stem cells, placental cells such as hAEC do not express substantial levels of telomerase, are not tumorigenic, and do not become aneuploid (Miki et al., 2005), thus being considered a promising source of stem cells. Indeed, several research groups have focused on the human placenta progenitor differentiation potential. Tamagawa et al. were the first to demonstrate pluripotent characteristics of cells from human amnion (Tamagawa et al., 2004). hAEC were shown to differentiate into all three germ layers, including hepatic, neural and pancreatic lineages (Miki et al., 2005; Miki and Strom, 2006). Naïve, undifferentiated cells of both amnion and chorion showed to express markers of glial and neuronal progenitor cells *in vitro* (Sakuragawa et al., 1996). Furthermore, cultured hAEC were found to produce acetylcholine, acetyltransferase and dopamine (Sakuragawa et al., 1997; Kakishita et al., 2000). Further, neuronal markers could be induced *in vitro* by exposure to retinoic acid in hAEC, hAMSC and hCMSC (Portmann-Lanz et al., 2006). Knezevic showed that ectopic *in vitro* transplantation of rat amnion under the kidney capsule resulted in differentiation into stratified squamous epithelium, a morphologically similar structure to skin, and derivatives, such as hair follicles and sebaceous glands (Knezevic, 1996). Mesodermal differential potential of hAMSC and hCMSC has also been demonstrated for both adipogenic and osteogenic lineages *in vitro* (In ‘t Anker et al., 2003). Other independent studies also reported the acquisition of chondrogenic, myogenic and cardiomyogenic fates (Wei et al., 2003; Miki et al., 2005; Portmann-Lanz et al., 2006; Ilancheran et al., 2007).

A capacity to commit to a functional phenotype has also been suggested *in vivo*. For example, hAEC transplanted into SCID/beige mice liver have been shown to integrate into the hepatic plate and adopt a hepatic phenotype, including secretion of albumin or α-1 antitrypsin, as well as metabolic features of functional hepatocytes (Sakuragawa et al., 2000; Marongiu et al., 2011). Differentiation of hAEC into lung epithelial cells was suggested in a bleomycin-induced lung injury model in immunodeficient mice, along with a distinct anti-fibrotic and anti-inflammatory effect was demonstrated (Moodley et al., 2010). Even pancreatic differentiation of hAEC was suggested *in vivo*, when transplantation of hAEC into the spleen of immunodeficient mice in a diabetic model restored normalized blood glucose levels in these animals; although also hAMSC were suggested to differentiate into the pancreatic lineage (Wei et al., 2003), the insulin production of the alleged hAMSC might actually be originating from a certain percentage of hAEC present in the culture (reviewed by Miki et al., 2005).

#### Immunomodulation

Together with the differentiation capabilities, different findings have demonstrated the reduced immunogenicity and multifaceted immunomodulatory capacity of placenta-derived cells. Within placenta tissues, hAMSC and their conditioned medium (hAMSC-CM) have been shown to reduce *in vitro* T cell proliferation induced by alloantigens, via T-cell receptor or mitogens (Magatti et al., 2008; Rossi et al., 2012). hAMSC and hAMSC-CM significantly reduce the expression of markers associated to Th1 and Th17 populations, and significantly induce the regulatory T cells compartment (Pianta et al., 2015, 2016). Amniotic cells are able to block the differentiation of monocytes into both dendritic cells (DC) and inflammatory M1-macrophages and to skew monocyte differentiation toward anti-inflammatory M2 macrophages. The macrophages generated in the presence of amniotic cells were poor inducers of T-cell proliferation, increased production of the anti-inflammatory cytokine IL-10, and reduced secretion of different pro-inflammatory factors (Magatti et al., 2009, 2015). Additionally, the macrophages generated in the presence of CM enhance wound healing in diabetic mice (Magatti et al., 2017). The therapeutic effects of amniotic cells and their secretome have been reported in other preclinical models of diseases based on inflammatory processes and with altered immune reactions, such as lung (Cargnoni et al., 2009, 2012, 2014) and liver fibrosis (Ricci et al., 2013; SantAnna et al., 2016; Cargnoni et al., 2017), sepsis, inflammatory bowel disease, autoimmune encephalomyelitis and rheumatoid arthritis (Parolini et al., 2014) cardiac ischemia (Cargnoni et al., 2009), and traumatic brain injury (Pischiutta et al., 2016). In these diseases, the modulation of inflammation seems to be a key element underlying the restoration of tissue integrity promoted by placental cells and their bioactive factors (Silini et al., 2017). Taken together, differentiation and immunomodulatory properties render placental cells, and their secretome, very interesting candidates for a variety of therapeutic applications, and in the following section their contribution to cardiac regeneration will be discussed.

### Placenta-derived cells in cardiac regeneration: mechanisms relevant for cardiac repair

The placental cells used in preclinical studies discussed in this section are hAMSC, hAEC, hCMSC, and hUCMSC (including Wharton's Jelly). In addition, MSC from the chorionic plate/trophoblast (hCpMSC), and MSC isolated from placenta tissue in toto (i.e., no specific compartment described; PDMSC) will be discussed. We will also consider studies in which Placenta-derived Adherent cells (PDA-001, Celgene Therapeutics), and PLacental eXpanded (PLX) mesenchymal-like adherent stromal cells (PLX-PAD, Pluristem Therapeutics Inc.) are used.

Essentially, there are 4 mechanisms that could be held accountable for the therapeutic effects mediated by placental cells on the injured cardiac tissue, which can be mainly related to their direct cell differentiation or to specific paracrine effects, such as anti-apoptotic, pro-angiogenic, and immunomodulatory properties as the result of the administration of their derivatives (i.e., the cell secretome as represented by their cell-conditioned medium and/or extracellular vesicles), respectively. These features make placental cells worthy competitors in the field of regenerative medicine. These will be discussed in the following sections in order to provide insight onto how placental cells and derivatives are able to support cardiac regeneration.

#### Cardiovascular and cardiomyogenic differentiation

Even if nowadays it does not seem to be the major mechanism supporting the placental-cell therapeutic effects (Balbi and Bollini, 2017) there is a modest amount of groups which have provided evidence of placental-cell differentiation toward cardiovascular and cardiomyocyte-like cells. Evidence of this is shown by the expression of cardiomyocyte markers and/or acquisition of spontaneous cell beating. *In vitro* placental cell differentiation has been suggested for UCMSC after incubation with 5-azacytdine (Pereira et al., 2008), and after co-culture of neonatal murine cardiomyocytes with hAMSC (Tsuji et al., 2010) and PDMSC (MSC isolated from placenta tissue in toto) (Liu et al., 2015). Alviano et al. reported *in vitro* angiogenic differentiation of hAMSC (Alviano et al., 2007), suggesting that term placenta might also contain progenitors with endothelial commitment potential, as reported by others (König et al., 2012, 2015; Meraviglia et al., 2012; González et al., 2015; Abumaree et al., 2017).

*In vivo*, even if in the majority of studies presented herein placental cells are few and rarely found in preclinical models at time of sacrifice, cardiomyocyte differentiation has been reported for hAEC following transplantation in immunocompromised rats with myocardial infarction (Fang et al., 2012). Likewise, PDMSC treated with a mixed cocktail of hyaluronan, butyric and retinoic acid (HBR) as cardiogenic/vasculogenic inducer, showed partial structural differentiation into cardiomyocyte-like cells, following transplantation into infarcted rat hearts (Ventura et al., 2007).

#### Cardioprotection and inhibition of apoptosis

Inhibition of cardiomyocyte apoptosis is pivotal in rescuing the cardiac tissue. Placental cells and their derivatives have been reported to be able to counteract cardiomyocyte apoptosis both *in vitro* and *in vivo* via significant paracrine influence. For example, conditioned media of UCMSC cultured in hypoxia has been shown to reduce apoptosis of cardiomyocytes (Santos Nascimento et al., 2014; Zhao et al., 2016), *in vitro*; as well, cardiac cell death was further reduced when secretome of HGF-transfected UCMSC (cultured in hypoxia) was used (Zhao et al., 2016). *In vivo*, the same group showed that transplantation of UCMSC was able to enhance cardiac function, decrease cardiomyocyte apoptosis, increase cardiomyocyte proliferation, and increase capillary density in immunodeficient mice with MI. These parameters were further improved following treatment with HGF-transfected UCMSC (Zhao et al., 2016). The anti-apoptotic effects of UCMSC have also been described by others following implantation in immune-competent mice with MI, whereby the number of apoptotic cells in infarcted hearts was reduced by 40% compared to vehicle-treated mice (Santos Nascimento et al., 2014). In addition, extracellular vesicles and exosomes isolated from UCMSC secretome have been shown to reduce cardiac fibrosis and cell apoptosis, while increasing cell proliferation in the hearts of rats with myocardial infarction, after intravenous injection (Zhao et al., 2015). Exosomes from UCMSC have also been studied by another group who observed a decrease of apoptotic cells in exosome-treated animals compared to PBS-treated ones (Ma et al., 2016). Furthermore, in a minipig model of MI, PDMSC-treated pigs showed lower TUNEL-positive cardiomyocytes at the infarct border area, compared to vehicle-treated pigs, 8 weeks after injury (Liu et al., 2015). Cardiomyocyte proliferation has also been shown to be enhanced after transplantation of placenta-derived adherent cells (PDA-001) in a mouse model of chronic heart failure (Chen et al., 2015). To further confirm the relevance of placenta cell cardioprotective paracrine potential, an innovative hydrogel formulation obtained from placental matrix and growth factors has been recently used to improve iPS-derived cardiomyocyte culture *in vitro*, also showing paracrine enhancement of cardiac repair as a delivery vehicle (Francis et al., 2017).

#### Pro-angiogenic effect

Regarding the pro-angiogeneic influence of perinatal progenitors, cell secretomes from both PDMSC (Liu et al., 2015) and PLX-PAD (Pluristem's mesenchymal-like adherent cells isolated from human term placenta) (Roy et al., 2013) have been shown to stimulate HUVEC proliferation and tube formation *in vitro*. In addition, exosomes from UCMSC were demonstrated to stimulate proliferation (Ma et al., 2016), migration (Zhao et al., 2015; Ma et al., 2016), and tube-forming ability (Zhao et al., 2015) of EA.hy926 human endothelial cells *in vitro*. Similarly, different types of placental cells have been shown to promote angiogenesis within the infarcted myocardium *in vivo*. For example, PDMSC significantly enhanced angiogenesis when injected into the MI border area in immune-compromised mice (Liu et al., 2015), resulting in increased vascularization compared to control group (Liu et al., 2015); improved heart contractibility was also detected in minipig hearts undergoing MI when PDMSC were injected in the border zone (Liu et al., 2015). Although functional cardiomyogenic differentiation of placental cells have been generally shown to be controversial, PDMSC were retained in the minipig hearts for up to 8 weeks post treatment while co-expressing HLA-ABC and cardiac troponin T, a marker for striated cardiomyocytes (Liu et al., 2015). In contrast, in a mouse model of myocardial infarction, chorionic plate MSC (CpMSC) did not successfully engraft and survive starting from third day post-injection, and cells disappeared more rapidly after the second and third injections (i.e., disappeared after 24 h), even if at 40 days after first treatment cardiac function was improved (Passipieri et al., 2014). In line with these results, transplantation of placenta-derived adherent cells (PDA-001) in a mouse model of chronic heart failure has been shown to increase endothelial cell proliferation and capillary density when injected intramyocardially (Chen et al., 2015). Notably, capillary density improvement was observed *only* when a low-dose (0.5 × 10^4^ vs. high dose 0.5 × 10^6^) of cells was used, suggesting the latter to be more efficient in providing tissue repair (Chen et al., 2015), despite no human cells were detected in the murine heart 28 days after injection (Chen et al., 2015). PLX-PAD have also been shown to stimulate arteriogenesis after injection in the peri-infarct area, following left anterior descending artery ligation in immune-competent mice (Roy et al., 2013), resulting into more abundant and mature arterial blood vessels. In addition, PLX-PAD-treated hearts showed higher microvessel density compared to vehicle-treated hearts. The same group also reported better cardiac regeneration capability of hAEC injected in mice with MI after their specific *in vitro* preconditioning in order to enhance epithelial-mesenchymal transition (EMT, i.e., hAEC treated with TGF beta for 6 days *in vitro* prior to injection), compared to non-induced hAEC (Roy et al., 2015). Indeed, hAEC sustained local angiogenesis, with greater resident cardiomyocyte pro-survival influence as exerted by EMT-hAEC (Roy et al., 2015).

The human amniotic membrane (AM) has also been shown to provide therapeutic effects when used as a patch implant in rodent models of cardiac disease. Cargnoni et al. have demonstrated that epicardial application of the AM patch on infarcted rat hearts is able to significantly reduce post-ischemic cardiac dimensional alterations, thus improving myocardial function (Cargnoni et al., 2009). Beneficial effects were apparent 7 days after AM application and continued for up to 60 days post treatment (Cargnoni et al., 2009). In line with this, Roy et al. revealed that treatment of infarcted heart with patches of either intact or decellularized amniotic membrane reduced infarct size (Roy et al., 2016). Decellularized AM showed to be immunologically inert, suggesting that ECM components in the AM might be crucial in exerting beneficial effects on the infarcted heart (Roy et al., 2016).

The pro-angiogenic effects of UCMSC have also been described following implantation in immune-competent mice with myocardial infarction (Santos Nascimento et al., 2014). In addition, exosomes from CM obtained from UCMSC have also been shown to increase blood vessel numbers in chick allantoic membrane assay (CAM), when compared to DMEM-treated CAM (Ma et al., 2016). Interestingly, exosomes obtained from Akt-induced UCMSC had a higher efficiency in promoting angiogenesis, and this was suggested to be due to the fact that Akt-induced exosomes shuttle PDGF (a growth factor found to be enriched in this type of exosomes) to recipient cardiac and endothelial cells, thus promoting their proliferation, migration, and blood vessel formation (Ma et al., 2016). The above-mentioned angiogenic properties along with the proposed paracrine mode of action of placental cells has encouraged several groups to investigate protein and soluble factor content in the conditioned medium of cultured placental cells. Pro-angiogenic proteins such as vascular endothelial growth factor (VEGF) and angiopoietin-1 have been shown to be found in PLX-PAD cell secretome, which were found increased when cell conditioned medium was collected from cells cultured in hypoxia (Roy et al., 2013).

In addition, hypoxia was able to decrease tissue inhibitor of matrix metalloproteinase-1, an inhibitor of endothelial cells (Reed et al., 2003) and promoter of myocardial fibrosis (Takawale et al., 2017). Others have also demonstrated that PDMSC can secrete hepatocyte growth factor (HGF), interleukin (IL)-8, and growth related oncogene (GRO)-alpha (Liu et al., 2015). MSC from fetal membranes can also secrete VEGF and HGF, which is enhanced when cells are treated with hyaluronan with butyric and retinoic acid (HBR) (Ventura et al., 2007). On another note, the same group showed that HBR also enhances *in vitro* differentiation of MSC toward cardiomyocyte-like cells, thus suggesting that HBF encompasses both differentiating features for MSC and potential to afford growth factor-mediated paracrine regeneration (Ventura et al., 2007). Likewise, conditioned medium from UCMSC cultured in hypoxia contains angiogenic factors such as VEGF, HGF, epidermal growth factor (EGF), and basic fibroblast growth factor (bFGF), and the presence of these factors is enhanced with CM is collected from HGF-transfected UCMSC cultured in hypoxia (Zhao et al., 2016).

#### Immunomodulatory potential

The inflammatory response to an injury is a critical regulator of the regenerative process. Immunomodulatory properties of placental cells have been widely demonstrated *in vitro* (Magatti et al., 2016) and transplantation of placental cells and their derivatives has been shown to exert *in vivo* therapeutic effects in a variety of diseases with altered immune responses (Magatti et al., 2016), thus supporting the notion that targeting inflammation is beneficial to the regenerative process (Silini et al., 2017).

When considering cardiac injury, placental cell immunomodulatory properties are a fertile ground for investigation and could potentially contribute to regeneration of the diseased heart. As in other immune-regulated diseases such as in lung (Cargnoni et al., 2009, 2012, 2014; Moodley et al., 2009, 2010; Murphy et al., 2011, 2012; Vosdoganes et al., 2011; Hodges et al., 2012; Chambers et al., 2014; Tan et al., 2014) and liver fibrosis (Tsai et al., 2009; Manuelpillai et al., 2010, 2012; Sant'Anna et al., 2011; SantAnna et al., 2016; Zhang et al., 2011; Ricci et al., 2013; Cargnoni et al., 2017), placental cells and their soluble derivatives have been shown to decrease fibrosis in the hearts of animals MI. Indeed, the inflammatory response after injury is critical in regulating tissue regeneration, and the inflammatory response occurs even after cardiac injury, thus what we learn from how placental cells modulate the inflammatory response to favor tissue regeneration in models of chronic fibrosis could shed light onto how placental cells could be beneficial to the regenerative process after cardiac injury. For example, transplantation of placenta-derived adherent cells (PDA-001) in a mouse model of chronic heart failure has been shown to improve cardiac performance and decrease fibrosis when injected intra-myocardially (Chen et al., 2015). Moreover, exosomes isolated from the CM of UCMSC have been shown to reduce cardiac fibrosis of rats with myocardial infarction after intravenous injection (Zhao et al., 2015). These studies instigate future ones in order to understand how the immunomodulatory properties placental cells and derivatives contribute to cardiac regeneration.

## Future perspectives in the adult and in the pediatric patient

Cardiovascular disease patients need prompt therapeutic intervention, especially pediatric ones affected by congenital heart disease.

**Figure 1:**
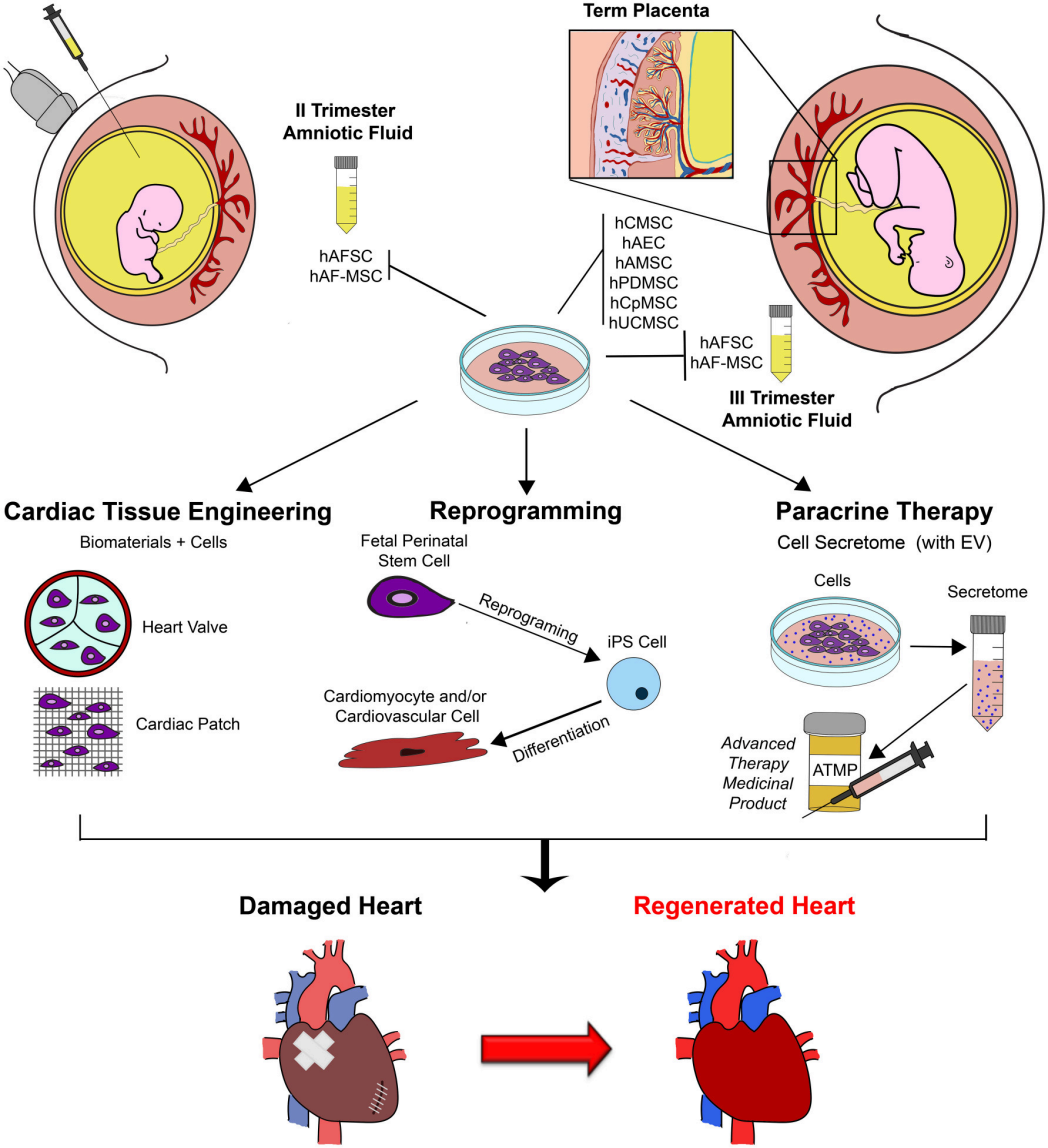
Schematic representation of exploitation of fetal perinatal stem cell biology for cardiac regeneration. Mesenchymal stromal progenitors can be isolated by amniotic fluid samples harvested during prenatal screening procedure (II trimester gestation) or at term, during scheduled cesarean delivery (III trimester); similarly, several population of stem cells can be isolated from term placenta at birth. Fetal perinatal stem cells can be easily isolated and cultured *in vitro*. Their peculiar regenerative features can be exploited to enhance cardiac repair and sustain cardiac regeneration by different approaches, including tissue engineering and cell therapy (possibly via perinatal stem cell reprogramming into more immature pluripotent cells to obtain mature cardiomyocyte and cardiovascular cells from) and paracrine therapy, via the formulation of the stem cell secretome, into a putative future advanced therapy medicinal product (ATMP). hAFSC, human c-KIT+ Amniotic Fluid Stem Cells; hAFS-MSC, human Amniotic Fluid Mesenchymal Stem Cells; EV, Extracellular Vesicles; hCMSC, human Chorionic Mesenchymal Stromal Cells; hAEC, human Amniotic Epithelial Cells; hAMSC, human Amniotic Mesenchymal Stromal Cells; hPDMSC, human Placenta-Derived Mesenchymal Stromal Cells; hCpMSC, human Chorionic plate/trophoblast Mesenchymal Stromal Cells; hUCMSC, human Umbilical Cord Mesenchymal Stromal Cells; iPS, induced Pluripotent Stem Cell.

In such scenario, the ideal stem cell source should be selected upon consideration their potential, feasibility of their isolation together, with their *in vitro* self-renewal properties. Thus, fetal and perinatal stem cells isolated either during pregnancy from left-over samples obtained for prenatal screening, or at term from clinical waste material, can offer added value compared to somatic adult sources, given their immature and developmentally “younger” potential, ease of availability, expansion and cryopreservation for long-term use while maintaining stable karyotype and low immunogenic profile. Furthermore these cells can also offer exclusive therapeutic advantages, as illustrated in the schematic Figure 1; indeed, on top their paracrine and differentiation potential, they can be reprogrammed more efficiently into pluripotent cells for putative drug-screening, cell therapy and cardiac tissue engineering approaches, and could be also envisioned for prenatal management and treatment of cardiac congenital diseases *in utero*, as already suggested by preliminary testing in preclinical animal models of different disease such as gastroschisis and spina bifida (Tam et al., 1989; Ramachandra et al., 2014; Dionigi et al., 2015; Shaw et al., 2016).

Nevertheless, further efforts should be made to address the standardization of cell isolation protocols and cell culture conditions, and also preparation of the cell secretome, since a definitive consensus has not been obtained yet; for sure, this represents a crucial aspect in order to avoid result diversification in preclinical studies and to support opportune clinical translation for future therapeutic strategies.

## Author contributions

SB: Supervised the work and mainly contributed to the discussion on mechanisms of cardiac repair and regeneration and on the amniotic fluid stem cell biology; CB: Contributed to critical discussion and assisted with the schematic figure; AS and OP: Contributed to the discussion on the mechanisms of action of placental cells in their therapeutic applications; SW and AB: Contributed by describing the basic properties of the placenta and the cells derived thereof. All authors reviewed the manuscript and approved it.

### Conflict of interest statement

The authors declare that the research was conducted in the absence of any commercial or financial relationships that could be construed as a potential conflict of interest.
